# Utilizing Technology for Diet and Exercise Change in Complex Chronic Conditions Across Diverse Environments (U-DECIDE): Protocol for a Randomized Controlled Trial

**DOI:** 10.2196/37556

**Published:** 2022-07-28

**Authors:** Riley C C Brown, Dev K Jegatheesan, Marguerite M Conley, Hannah L Mayr, Jaimon T Kelly, Lindsey Webb, Amandine Barnett, Heidi M Staudacher, Nicola W Burton, Nicole M Isbel, Graeme A Macdonald, Katrina L Campbell, Jeff S Coombes, Shelley E Keating, Ingrid J Hickman

**Affiliations:** 1 School of Human Movement and Nutrition Sciences The University of Queensland Brisbane Australia; 2 Department of Nephrology Princess Alexandra Hospital Brisbane Australia; 3 Faculty of Medicine The University of Queensland Brisbane Australia; 4 Department of Nutrition and Dietetics Princess Alexandra Hospital Brisbane Australia; 5 Nutrition and Dietetics Research Group Bond University Robina Australia; 6 Centre for Online Health The University of Queensland Brisbane Australia; 7 Centre for Health Services Research The University of Queensland Brisbane Australia; 8 Food & Mood Centre Institute for Mental and Physical Health and Clinical Translation Deakin University Melbourne Australia; 9 School of Applied Psychology Griffith University Brisbane Australia; 10 Department of Gastroenterology and Hepatology Princess Alexandra Hospital Brisbane Australia; 11 Healthcare Excellence and Innovation Metro North Health Brisbane Australia; 12 Menzies Health Institute Queensland Griffith University Brisbane Australia

**Keywords:** lifestyle intervention, telehealth service delivery, digital disruption, complex chronic disease, liver disease, kidney disease, transplant, metabolic syndrome, metabolism, diabetes, obesity, mobile health, health technology, chronic disease

## Abstract

**Background:**

The metabolic syndrome is common across many complex chronic disease groups. Advances in health technology have provided opportunities to support lifestyle interventions.

**Objective:**

The purpose of this study is to test the feasibility of a health technology-assisted lifestyle intervention in a patient-led model of care.

**Methods:**

The study is a single-center, 26-week, randomized controlled trial. The setting is specialist kidney and liver disease clinics at a large Australian tertiary hospital. The participants will be adults with a complex chronic condition who are referred for dietetic assessment and display at least one feature of the metabolic syndrome. All participants will receive an individualized assessment and advice on diet quality from a dietitian, a wearable activity monitor, and standard care. Participants randomized to the intervention group will receive access to a suite of health technologies from which to choose, including common base components (text messages) and optional components (online and mobile app–based nutrition information, an online home exercise program, and group-based videoconferencing). Exposure to the optional aspects of the intervention will be patient-led, with participants choosing their preferred level of engagement. The primary outcome will be the feasibility of delivering the program, determined by safety, recruitment rate, retention, exposure uptake, and telehealth adherence. Secondary outcomes will be clinical effectiveness, patient-led goal attainment, treatment fidelity, exposure demand, and participant perceptions. Primary outcome data will be assessed descriptively and secondary outcomes will be assessed using an analysis of covariance. This study will provide evidence on the feasibility of the intervention in a tertiary setting for patients with complex chronic disease exhibiting features of the metabolic syndrome.

**Results:**

The study was funded in 2019. Enrollment has commenced and is expected to be completed by June 2022. Data collection and follow up are expected to be completed by December 2022. Results from the analyses based on primary outcomes are expected to be submitted for publication by June 2023.

**Conclusions:**

The study will test the implementation of a health technology–assisted lifestyle intervention in a tertiary outpatient setting for a diverse group of patients with complex chronic conditions. It is novel in that it embeds patient choice into intervention exposure and will inform health service decision-makers in regards to the feasibility of scale and spread of technology-assisted access to care for a broader reach of specialist services.

**Trial Registration:**

Australian New Zealand Clinical Trial Registry ACTRN12620001282976; https://www.anzctr.org.au/Trial/Registration/TrialReview.aspx?id=378337

**International Registered Report Identifier (IRRID):**

DERR1-10.2196/37556

## Introduction

The metabolic syndrome (MetS) is a combination of clinical risk factors, including central obesity, hypertension, dyslipidemia, and hyperglycemia [1]. Presence of the MetS is associated with increased risk of cardiometabolic complications, including cardiovascular disease and type 2 diabetes [2]. On a global scale, the MetS represents a substantial treatment burden due to increased costs and medication expenditure, hospitalization, and utilization of outpatient services [3,4]. The prevalence of the MetS is up to 60% in tertiary health care settings among patients with complex chronic conditions, including kidney and liver disease [5-7].

Specific components of the MetS may warrant pharmacotherapy, but this should only occur on a background of lifestyle intervention with a focus on diet quality and exercise [8-11]. Previous diet and exercise interventions in people with chronic disease have shown a cardioprotective effect [12-18]. However, the implementation of community-based diet and exercise management programs for complex chronic diseases has traditionally been challenging. Patients with chronic kidney or liver disease often have multimorbidity, potentially further compounding metabolic risk [19,20]. Management has traditionally required contact with multiple specialist teams across a siloed system, creating a significant burden for patients and caregivers [17,21]. Diet and exercise services have traditionally been delivered in condition-specific specialist settings, often with insufficient resources to deliver adequate services to address the health issues [22-24]. An integrated approach for the delivery of exercise and diet interventions through a unified complex chronic disease model of care could support improvement of cardiometabolic risk in tertiary care across specialist groups.

Advances in health technology have provided new opportunities to assist lifestyle interventions and improve the management of the MetS [13,17]. Asynchronous (eg, online information and resources) and synchronous (eg, telehealth appointments with health professionals) technology interventions have emerged as viable options for delivery of health care. These have the potential to address current barriers to service delivery, particularly in rural areas, including continuity of care, availability of resources, and location of services [25]. A range of factors within complex tertiary care systems, including patient-related factors (eg, values, goals, motivations, skills, and knowledge), organizational-related factors (eg, culture, priorities, and structural systems), and external factors (eg, health care funding), must be considered when assessing the feasibility of disruptive service change [26]. Patient-led approaches allow for the patient’s perspective to be considered when receiving health care [27]. These approaches ideally assist patients in individualizing their self-management plans through selecting which services they want to engage with and through what means.

This study will expand on the current literature by testing the implementation of a health technology–assisted lifestyle intervention for people with complex chronic disease. The primary aim will be to test the feasibility of this intervention, determined by safety, recruitment rate, retention, exposure uptake, and telehealth adherence. The secondary aims include assessing the clinical effectiveness of the intervention, (including metabolic syndrome severity, dietary quality, physical activity and sedentary behavior, exercise capacity, neuromuscular fitness, muscular pain, clinical parameters, quality of life, perceived confidence, fatigue, and sleep quality and quantity), patient-led goal attainment, treatment fidelity, exposure demand, and participant perceptions. We hypothesize that delivery of lifestyle interventions using health technology is feasible, can address the barriers to service delivery, and will demonstrate improved clinical outcomes over standard care.

## Methods

### Study Design and Setting

**Figure 1:**
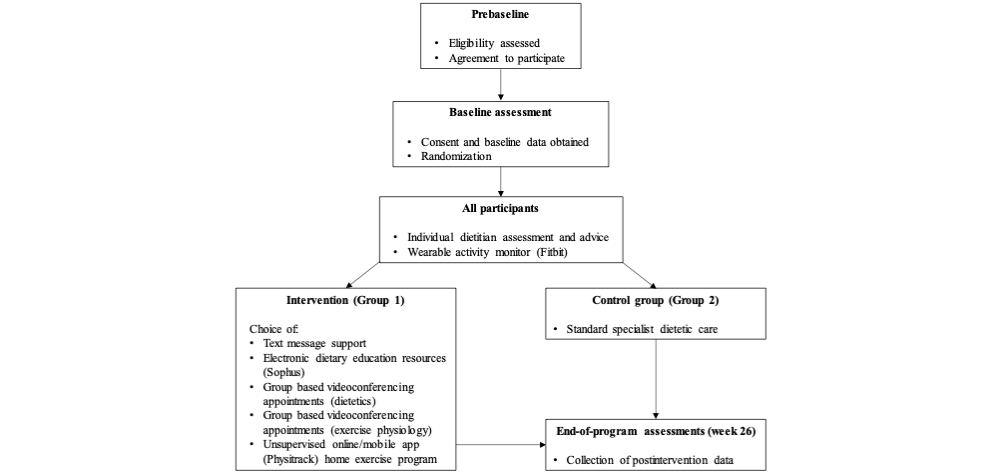
Study design and participant flow, indicating the base components offered to all participants and the additional technology-assisted components offered to the intervention group.

This is a single-center, 26-week, randomized controlled trial. The study is being conducted in a public hospital in a major metropolitan city in Queensland, Australia. The study design and flow are shown in Figure 1.

### Participants and Eligibility Criteria

Adults living with kidney or liver disease who are at increased cardiometabolic risk and receiving specialist care at the Princess Alexandra Hospital in Brisbane, Australia, will be included. Eligibility criteria are shown in Textbox 1.

**Eligibility criteria.**

**Inclusion criteria**

- Under the outpatient care of at least one of the following specialist Princess Alexandra Hospital clinics: kidney or liver transplant, chronic kidney disease, hemodialysis, peritoneal dialysis, or hepatology
- Have (or be undergoing treatment for) at least one of the features of the metabolic syndrome, as defined by the harmonized criteria [1]:
  Elevated blood pressure (or on medication to treat) (systolic blood pressure ≥130 mm Hg and/or diastolic blood pressure ≥85 mm Hg)
  Elevated waist circumference (population- and country-specific definitions [1])
  Reduced high-density lipoprotein cholesterol (or on medication to treat) (<1.0 mmol/L in males; <1.3 mmol/L in females)
  Elevated fasting blood glucose (or on medication to treat) (≥5.6 mmol/L)
  Elevated triglycerides (or on medication to treat) (≥1.7 mmol/L)
- Deemed suitable to participate by treating medical specialist
- Screened as capable to participate by an exercise professional
- Have current access to a mobile device or computer hardware with internet access and webcam capability

**Exclusion criteria**

- Non–English speaking or unable to read and write in English
- Documented malnutrition
- Under 18 or over 80 years of age
- Currently pregnant or breastfeeding
- Life expectancy less than 6 months

### Recruitment

The recruitment target will be 168 participants. Potentially eligible patients will be sourced via screening referrals to the dietetics department from a medical specialist. Referrals will be initially screened by the research project officer, and potentially eligible people will be contacted by phone to complete eligibility screening and be invited to participate. If a patient agrees to participate, a baseline appointment will be scheduled by the research project officer. Participant information and consent forms will be provided to patients via email prior to the baseline assessment, and written informed consent will be obtained at the baseline appointment. Age, sex, referring clinic, and inclusion and exclusion information for patients deemed ineligible or who decline to participate will be collected from electronic medical records. Ineligible patients will continue with the process of standard dietetic care as per usual practice guidelines. Demographic and medical data (Table 1) will be collected via online forms, review of medical charts, and phone calls prior to the baseline assessment. This is deemed a low-risk study, with expectations of behavior change no different from current usual care.

### Randomization and Allocation

Participants will be randomized 1:1 to the intervention group (group 1) or to a control group (group 2) that will receive standard care and individualized dietetic advice to support improvements in diet quality [28]. Computer-generated randomization will be completed using the REDCap research management system [29]. Groups will be stratified by referral source: (1) chronic kidney disease clinic, (2) hepatology clinic, or (3) liver or kidney posttransplant clinic. Participants will be informed of their group allocation following baseline assessment by the research project officer.

### Assessors and Blinding

Baseline assessments will occur prior to randomization. Assessors performing end-of-program clinical assessments will be blinded to group allocations. Assessor blinding will be verified by questionnaire at time of completion for each participant (Likert scale 1-5). This questionnaire will evaluate whether assessors have remained blinded to participant allocation. Participants will be reminded by the research project officer prior to end-of-program assessments to not disclose their group allocation to assessors.

### Quantitative Data Collection

Baseline demographic and medical history data that will be collected are listed in Table 1. Research outcomes and the associated measurement methods are outlined in Table 2. Primary outcomes will be assessed once all participants have finished the trial, and secondary outcomes will be collected at baseline and end-of-program assessments. Once a participant is randomized, the research staff will make every reasonable effort (eg, via phone calls, text messages, and emails) to follow the participant for the entire study period for collection of data.

**Table 1:** Demographic and medical data collected prior to the baseline assessment.

Demographic and medical data	Measurements
Sex	Male or female
Date of birth	Date
Ethnicity	Caucasian, Indigenous, European, Anglo-Saxon, Asian, other, unknown/not reported
Marital status	Single or never married, living together, de facto or married, separated or divorced, widowed
Highest education level completed	Primary school, less than grade 10, grade 10, grade 12, vocational school or college, university
Employment status	Full-time, part-time, unemployed, self-employed, student, retired
Medication use	Listed medication intake including type, dose, and frequency
Menopause status	Yes, no, not applicable
Allergies	Yes, no, listed allergies
Cigarette smoking history	Former, current, never
Alcohol consumption	Number of standard drinks per week
Need for assistance to read written health materials^a^	Never, rarely, sometimes, often, always
eHealth literacy	eHealth Literacy Scale (eHEALS)^b^ questionnaire [30]

^a^Assessed through the following question via online form: “How often do you need to have someone help you when you read instructions, pamphlets, or other written material from your doctor or pharmacy?”

^b^eHEALS is a validated 8-question digital health literacy questionnaire that evaluates the ability to find the right type of health information online, where and how to find and use it, possession of the skills and confidence to evaluate the quality of online health information, and the perception of the usefulness and importance of accessing online information for health [30]. This will be administered via an online form.

**Table 2:** Primary and secondary outcomes and their associated measurements.

Outcomes					Measurements
**Primary outcomes**					
	Safety			Number of study-related serious adverse events	
	Recruitment rate			Number of patients recruited as a proportion of all referred eligible patients	
	Retention			Number of intervention participants undergoing end-of-program assessment	
	Exposure uptake			Frequency of dietetic and exercise specialist contact (within public hospital system) as a proportion of total scheduled contacts	
	Telehealth adherence			Attendance to online exercise and dietetic sessions as a proportion of total scheduled contacts	
**Secondary outcomes**					
	**Clinical effectiveness**				
		Metabolic Syndrome Severity Score [31]	Algorithm score comprising systolic blood pressure, diastolic blood pressure, waist circumference, triglycerides, high-density lipoprotein cholesterol, and fasting blood glucose		
		Dietary quality	Diet quality assessed by food group intake (frequency per day or week), fiber intake (grams per day), unsaturated oils (grams per day), and discretionary food intake (grams per day)		
		Physical activity and sedentary behavior	Time (intensity-weighted minutes) spent in past week as assessed by International Physical Activity Questionnaire (Short Form) and Fitbit weekly physical activity heart rate data		
		Exercise capacity	6-Minute Walk Test score		
		Neuromuscular fitness	Single chair stand, Five Times Sit to Stand test, hand grip strength		
		Muscular pain	Modified Nordic Musculoskeletal Questionnaire		
		Quality of life	European Quality of Life Five Dimension Five Level Scale		
		Nutrition and physical activity management self-efficacy	Likert confidence scales		
		Fatigue	Functional Assessment of Chronic Illness Fatigue Scale score		
		Sleep quality and quantity	Pittsburgh Sleep Quality Index score, total Fitbit weekly sleep data		
		Additional clinical parameters	Resting heart rate, BMI, serum biochemical analytes (Multimedia Appendix 1)		
	Goal attainment			Goal Attainment Scale score	
	Treatment fidelity			Treatment notes^a^ Staff attendance at professional support and case discussions	
	Exposure demand			Selection of health technology options	
	Participant perceptions			Feedback survey Qualitative individual interview data (subsample)	

^a^Participant session notes taken by health professionals from telehealth-facilitated sessions with the intervention group.

### Primary Outcome

The primary outcome is feasibility (Table 2), which will be used to inform future service delivery. Feasibility success will be confirmed if the intervention is safe (defined as a similar number of study-related serious adverse events [SAEs] between groups) and if three of the following four criteria are fulfilled: (1) ≥50% of all referred eligible patients are recruited (recruitment), (2) ≥70% of intervention participants undergo an end-of-program assessment (retention), (3) ≥75% of intervention participants have a higher frequency of specialist outpatient dietetic and exercise specialist contact than the controls (exposure uptake), and (4) videoconferencing-facilitated dietetic and exercise sessions have an attendance rate of ≥80% of the total scheduled contacts (telehealth adherence).

#### Safety

Safety will be determined from adverse event reporting. SAE categories and adverse events of special interest are detailed in Textbox 2. SAEs will be classified by event category, the outcome of the event, the relationship to the study, and by whether they were planned or unplanned. The relationship of the SAE to the study will be determined by medical review. All adverse events will be monitored and recorded via the REDCap research management system throughout the study [29]. In addition, all participants will be asked by a member of the research staff at the end-of-program assessment whether any unreported SAE or adverse event had occurred during the study period.

**List of serious adverse events and adverse events of special interest to be recorded and monitored during the study period.**

**Serious adverse event categories [32]**

- Death
- Life-threatening event
- Hospitalization (acute or prolonged), including planned admissions
- Event resulting in persistent or significant disability or incapacity
- Important medical event (eg, adverse drug reaction)
- Pregnancy

**Adverse events of special interest [32]**

- Hypoglycemic episode requiring assistance from another individual
- Fall
- Musculoskeletal injury requiring medical or allied health attention
- Hyperkalemia (newly diagnosed or requiring intervention)
- Episode of low blood pressure requiring medical attention
- Chest pain requiring medical attention

### Secondary Outcomes

The secondary outcomes that will be evaluated are clinical effectiveness (including metabolic syndrome severity, dietary quality, physical activity and sedentary behavior, exercise capacity, neuromuscular fitness, muscular pain, clinical parameters, quality of life, nutrition and physical activity self-efficacy, fatigue, and sleep quality and quantity), patient-led goal attainment, treatment fidelity, exposure demand, and participant perceptions (Table 2).

#### Clinical Effectiveness

All clinical effectiveness data will be collected at baseline and end-of-program assessments.

##### Metabolic Syndrome Severity Score

The metabolic syndrome severity score (MetSSS) is a continuous risk assessment score for quantifying severity of the MetS. It will be calculated using an algorithm developed from an Australian population that includes systolic blood pressure, diastolic blood pressure, waist circumference, and fasting blood measures (triglycerides, high-density lipoprotein cholesterol, and glucose) [31].

##### Dietary Quality

Habitual dietary intake of the participants will be collected via a 3-day self-administered diet record (2 weekdays and 1 weekend day) using the Research Food Diary mobile app (Xyris Software [Australia] Pty Ltd) [33]. Participants will be asked to record information on the type, portion, and brand of all consumed foods. This includes condiments, oils, herbs and spices, and beverages. The app is blinded such that participants cannot access any nutrient or composition information of foods. Participants will be asked to send their diet record data via the email function, and it will be verified via phone by a study dietitian before analysis. FoodWorks 10 Professional (Xyris) will be used for nutritional analysis of the diet records using the AusFoods 2019 and AusBrands 2019 databases, including estimated intakes of energy, macronutrients, micronutrients, and food groups [34]. Dietary intake data will be used to compare dietary quality of both study groups at baseline and end-of-program assessments. This includes servings or grams per day of food groups (fruit, vegetables, whole grains, legumes, nuts and seeds, and fish and seafood), fiber, unsaturated oils, and discretionary foods (processed meats, solid fats, added sugars, and alcoholic drinks). To minimize inaccurate self-reporting, participants with implausible intakes of <500 or >3500 kcal/day will be excluded [35].

##### Physical Activity and Sedentary Behavior

The International Physical Activity Questionnaire Short Form (IPAQ-SF) [36] will be used to assess self-reported behavior in the previous week. The survey will be administered by a health professional with experience in physical activity research in an interview setting. Participants will be guided through the questionnaire to gain an understanding of how much time they spend per week being sedentary and performing different intensities of physical activity. IPAQ-SF results will be reported in metabolic equivalent (MET) minutes per week of total activity and minutes per week for sedentary behavior, moderate physical activity, and vigorous physical activity. Heart rate (HR) data from a wearable activity monitor will be used to objectively measure weekly minutes of moderate- to vigorous-intensity physical activity throughout the study. The monitor allows for this intensity range to be preset in a custom heart rate zone. Participants will be provided with information about how to use and synchronize the device at the baseline assessment.

##### Exercise Capacity

The 6-Minute Walk Test (6MWT) will be used to assess exercise capacity. The test will be conducted indoors with temperature control on a 20-meter track under the supervision of a health professional. It will be conducted according to a standardized protocol [37]. The result of the 6MWT will be distance walked, rounded to the nearest meter.

##### Neuromuscular Fitness

The single and repeated chair stand tests will be used to assess functional lower limb neuromuscular strength and endurance. The single chair stand test requires the participant to start in a seated position with the hands crossed against the chest, stand, and then return to a seated posture as quickly as possible. The repeated chair stand test records the time it takes for the participant to complete 5 stands (5xSTS) [37]. One trial will be allotted for this test. The hand grip strength test will be completed using a grip dynamometer 3 times for each hand, with the highest result recorded for both the dominant and nondominant hand. All tests will be conducted under the supervision of a health professional, according to standardized protocols [37].

##### Muscular Pain

Participants will complete the Modified Nordic Musculoskeletal Questionnaire (MNMQ) [38] to quantify musculoskeletal pain. This will be administered via an online form provided by email prior to the baseline and end-of-program assessments.

##### Clinical Parameters

Body mass and height will be measured by a member of the research team with a calibrated weight scale and stadiometer. BMI will be calculated for each participant. Duplicate waist circumference will be measured at a level midway between the lower rib margin and iliac crest in the horizontal plane [37], and the mean will be recorded. If there is a greater than 1.5% difference between waist measures, a third measure will be taken and the median will be recorded [37]. Resting heart rate, systolic blood pressure, and diastolic blood pressure will be measured. Triplicate blood pressure and heart rate measurements will be taken approximately 2 minutes apart, with the participant in a seated position and having rested for at least 10 minutes. Participants will be asked when they last took medication or consumed caffeine. The first blood pressure measurement will be discarded and the mean of the last 2 readings will be used. Biochemical blood analytes will be measured at accredited pathology laboratories (Multimedia Appendix 1).

##### Quality of Life and Self-Efficacy

The EQ-5D-5L scale will be administered via online survey prior to physical assessments and will be used to evaluate quality of life [39]. Four-point validated Likert scales, ranging from 1 (very uncertain) to 4 (very certain), will be used to assess self-efficacy in overcoming barriers related to eating healthy food and carrying out exercise intentions and will be administered under the supervision of a health professional [40].

##### Sleep Quality and Quantity and Fatigue

The Pittsburgh Sleep Quality Index (PSQI) survey will be administered via online survey prior to in-person assessments to quantify sleep quality and duration [41]. Weekly sleep data will be collected using a wearable activity monitor. The monitor records total sleep time and time in sleep stages (deep, light, rapid eye movement, and wakefulness). Participants will be asked to wear the monitor during the night to capture sleep data. The Functional Assessment of Chronic Illness Fatigue Scale (FACIT; version 4) [42] will be administered via online survey prior to in-person assessments to evaluate fatigue.

#### Goal Attainment Score

Goal setting will be facilitated at the baseline assessment for all participants. Participants will be encouraged to set SMART (specific, measurable, achievable, realistic, timely) goals [43]. The goal attainment scaling method will be used to compare goal attainment between the intervention and control groups at end-of-program assessments [44].

#### Treatment Fidelity

Treatment fidelity will be assessed throughout the trial across three areas: training provided, delivery of treatment, and receipt of treatment [45]. Attendance of clinical staff at standardized orientation training and refresher training sessions, as well as regular professional support and case discussions, will be recorded. For delivery of dietetic treatment, the clinicians will summarize the topics covered at the end of each group telehealth session (eg, healthy snacks, vegetables and fruits, and legumes) and the frequency of topics and themes discussed over the course of the project will be described. For delivery of exercise treatment, a record of each training session prescription will be captured.

#### Exposure Demand

Exposure demand will be assessed descriptively by summarizing the frequency of each technology option chosen by the intervention group at baseline and summarizing the different combinations of technologies chosen by the intervention group at baseline.

#### Participant Perceptions

Quantitative and qualitative feedback from participants will be collected at end-of-program assessments. Prior to assessment day, participants will be asked to complete an online questionnaire using a 5-point Likert scale to assess the acceptability of different aspects of the intervention received, its usability, and their satisfaction with it. Quantitative data from the surveys will be analyzed using simple descriptive statistics.

A subsample of participants from both groups will be deliberately selected to achieve maximum demographic diversity for age, sex, and disease group, and they will be invited to provide feedback regarding the model of care received. Semistructured one-on-one interviews will be conducted by a health professional with experience in qualitative research training and no role in delivering the service model. We will interview the participants in-person at the end-of-program assessment visit or via telephone at a time convenient to the participant. Questions will be designed to capture the participant’s impressions of the service model they experienced; potential barriers and facilitators to the diet and exercise recommendations; their perspectives on the technology-assisted delivery methods, usability, and acceptability; and their self-reported intentions for future use of diet monitoring technology after completion of the study period. All interviews will be audio recorded, transcribed verbatim, and thematically analyzed with an iterative and inductive approach.

### All Participants

All participants will receive usual medical and specialist care and may choose to discontinue contact with the health professionals at any time during the study period. All participants will be offered an individualized in-person appointment with a dietitian, review appointments, and a wearable activity monitor. Patients do not currently have access to exercise physiology services as part of standard care.

#### Individual Dietitian Assessment and Advice

All participants will receive a personalized nutrition assessment and dietary advice aligned with principles of healthy eating for reducing cardiometabolic risk [28]. These principles are informed by evidence-based dietary patterns, including Mediterranean-style and DASH (dietary approaches to stop hypertension) diets [28], and focus on eating fruit, vegetables, whole grain cereals, healthy protein sources (especially fish, legumes, nuts, and seeds), unflavored dairy foods, healthy fats and oils (including extra virgin olive oil), and the use of herbs and spices instead of salt. This advice will be provided by an accredited practicing dietitian (APD) in an in-person appointment. The advice will be tailored to personal preferences and goals.

#### Wearable Activity Monitor

All participants will be provided with a Fitbit Inspire HR (Fitbit, Inc) device and its accompanying mobile app to monitor physical activity. The heart rate reserve (HRR) method will be used to quantify moderate-to vigorous-intensity physical activity (40-89% HRR), with resting heart rate measured at baseline and maximal heart rate estimated as follows: 208 – (0.7 × age) [46]. For participants taking beta-blocker medication, which affects heart rate, a more representative equation will be used: 168 – (0.51 × age) [47]. At the baseline assessment, participants will be instructed on how to use the Fitbit, including how to download the app and synchronize the device, and will be provided with an information booklet. Each participant will have a Fitbit account established with a study-specific email address that will be accessible to study staff. This will permit appropriate real-time data extraction for the study. If no device data is present for 7 days during the study period, participants will be contacted via text or phone call to troubleshoot. Participants will keep the device after the end-of-program assessments.

### Intervention

Participants randomized to the intervention group will be offered access to a suite of health technology options with both core (text message support) and optional (electronic dietary education resources, group-based exercise physiology and dietetics videoconferencing appointments, and an unsupervised online or mobile app home exercise program) components. Exposure to the frequency of text messages and the number and type of optional components will be patient-led, with participants choosing their preferred level of engagement (Figure 1). Participants will choose their components after randomization and the research project officer will confirm their choice via phone call in week 1. The core and optional components are described in the following sections.

#### Text Message Support

All intervention participants will receive lifestyle-related text messages. The text messages will be semipersonalized and unidirectional (Table 3). They will reflect selected behavior change techniques to help facilitate change in dietary and physical activity patterns [48,49]. Participants will be able to select the desired frequency of text messages: once, twice, or three times per week for the duration of the intervention.

**Table 3:** Text messages and relation to behavior change technique constructs.

Behavior change technique constructs	Text message examples
Social support	“Telling your helpful family and friends about your goals will help you achieve them”
Prompt specific goal setting	“2 serves of fruit every day is an important goal”
Behavior substitution	“Swapping 2 red meat meals for fish is a great way to get healthy sources of protein and fats!”
Prompt self-monitoring of behavior	“Monitor your resistance and aerobic exercise training sessions using the Fitbit watch and app”
Provide information about behavior-health link	“Resistance exercise twice a week increases muscle size and strength, helping you lose weight and keep healthy”
Problem solving	“Are you finding it hard to get 30 minutes of physical activity every day? Start with a smaller amount and build from there. Every little bit helps!”
Provide instruction	“To get more fiber from fruit, keep the skin on and aim for 2 serves per day”

#### Option 1: Electronic Dietary Education Resources

Participants can choose to have access to online and mobile app–based nutrition information from Sophus (Sophus Health Pty Ltd). These include nutrition fact sheets, nutrition information videos, personal journals, and recipes promoting the principles of healthy eating for reducing cardiometabolic risk [28].

#### Option 2: Group Based Videoconferencing Appointments (Dietetics)

Participants can choose to have access to group dietitian telehealth sessions (maximum 6 participants per group) via the Queensland Health telehealth portal. Sessions will be run by an APD and will be offered monthly. Sessions will last for 45 minutes and focus on principles of healthy eating for reducing cardiometabolic risk.

#### Option 3: Group Based Videoconferencing Appointments (Exercise Physiology)

Participants can choose to have access to group exercise telehealth sessions (maximum 6 participants per group) via the Queensland Health telehealth portal. Sessions will be run by an accredited exercise physiologist (AEP) and will be offered weekly. Sessions will last for 60 minutes. Participants will also be offered a personalized home exercise program prescribed through Physitrack (Physitrack Ltd, London, UK).

#### Option 4: Unsupervised Online and Mobile App Home Exercise Program

Participants can choose to access an unsupervised personalized home exercise program prescribed through Physitrack. This option does not include exercise telehealth sessions with an AEP. Home programs will be individually updated monthly by an AEP.

#### Exercise Intervention

Further details for the exercise intervention can be seen in Multimedia Appendix 2. In brief, participants opting into one of the two exercise options will be asked to achieve a minimum of 150 minutes of moderate- to vigorous-intensity aerobic exercise, and complete two 30-minute resistance exercise sessions per week. Participants who choose either exercise option will be provided with equipment (resistance bands with light and medium resistance grades) to facilitate resistance exercise. The prescribed videoconferencing exercise sessions will consist of 20 minutes of aerobic exercise, 30 minutes of resistance exercise, and 5-minute warm ups and cool downs. The AEP will remind the participants of their target heart rate zone and ask them to monitor their heart rate during the training session with their wearable activity monitor. The repetitions in reserve (RiR) method will be utilized for prescribing resistance exercise intensity via autoregulation [50]. Autoregulation allows resistance training to be adjusted in response to an individual’s performance in the session [51]. The prescribed exercise sessions will include 2 to 4 sets of a load between 1 to 4 RiR utilizing the provided equipment or body weight exercises. Participants who choose either exercise option will be provided with a personalized home exercise program following the same plan as the videoconferencing sessions.

### Statistical Analysis

#### Sample Size

As this will be a feasibility study, a sample size calculation was not performed for the primary outcome. To allow for reliable findings, the sample size calculation was conducted using the MetSSS for clinical effectiveness as a secondary outcome. Assuming a correlation of 0.5 between baseline and 26-week MetSSS and an effect size of 0.42 (representing a change of 0.8 in the intervention group and no change in the control group, with a pooled standard deviation of 1.9), the Stata procedure “sampsi” suggests that 67 participants per arm will be required to achieve 80% power to detect a significant difference at the 5% level (2-sided) using an analysis of covariance (ANCOVA). Allowing for 20% dropout, the required number of participants to be recruited will be 168 (84 per arm).

#### Primary Analysis

The primary analysis will evaluate the feasibility of the intervention. As mentioned previously, the feasibility of the intervention will be determined by evaluating safety and if three of the following four criteria are achieved: (1) recruitment rate ≥50%, (2) retention ≥70%, (3) exposure uptake ≥75%, and (4) telehealth adherence ≥80%. Safety will be assessed by comparing the number of study-related SAEs in the intervention and control groups using a chi-square test of homogeneity. The safety of the intervention will be assumed if there are no statistical differences between the groups.

#### Secondary Analyses

Analysis for continuous clinical outcomes will be conducted via an ANCOVA to compare change values between study groups, adjusted for baseline values and patient population (ie, patients with chronic kidney disease, hepatology, and kidney and liver posttransplant). No imputation of missing values will occur.

Supplementary descriptive analysis of participant technology choices and patient-led goal attainment will be conducted. Summative statistics for the proportion of engagement among participants in the intervention arm will be collected and analyzed postintervention. A significance level of *P*<.05 will be used for all analyses.

#### Patient and Public Involvement

Significant consumer engagement was undertaken in the design of the research question and health priorities; this involved focus groups and interviews [21,24,49,52,53]. This informed the design of the intervention, particularly the inclusion of patient choice in how to engage with technology and the acceptability of using telehealth options for diet and exercise support. Patients were not involved in the recruitment or conduct of the study and did not assess the burden of the intervention. Written summaries of the results will be sent to participants upon completion of the study.

#### Ethics Approval

This trial has been approved by the Metro South Human Research Ethics Committee (HREC/2019/QMS/58285) and The University of Queensland Human Research Ethics Committee (2020000127). The trial adheres to the Helsinki declaration and has been prospectively registered with the Australian New Zealand Clinical Trial Registry (ACTRN12620001282976).

## Results

The study was funded in 2019 and has been approved by the Metro South Human Research Ethics Committee and The University of Queensland Human Research Ethics Committee. Due to the COVID-19 pandemic, study enrollment was delayed for 6 months. Participant enrollment has commenced and will continue through June 2022. End-of-program assessments will continue through December 2022, when data collection is expected to be completed. Analysis, interpretation, and preliminary dissemination of results are planned for January 2023 to June 2023 through scientific conferences. The main results are expected to be published by August 2023.

## Discussion

This trial will test the implementation of a technology-assisted lifestyle intervention for a diverse group of patients with complex chronic conditions who receive specialist outpatient care from the tertiary health system. While telehealth delivery of dietary interventions has been shown to be cost-effective in research settings [54], this mixed methods approach incorporates feasibility and effectiveness outcomes, as well as patient engagement strategies to evaluate service redesign in a real-world clinical setting. While trials of technology such as telephone coaching or video-enabled telehealth counselling sessions have generally been deemed feasible for some complex conditions [13,48], patients’ desire for greater choice and to lead decision-making around engagement with a diverse suite of technologies is clear [21,52]. We expect that the intervention will be able to address a number of barriers to engaging with lifestyle interventions in the hospital and health care system, and that it will allow improved access to specialist care compared to standard practice. The results of this project will be of interest to researchers and policy makers who are strategically investing in a transition to telehealth models of care. This is especially pertinent in the context of the global COVID-19 pandemic, which challenged traditional health care delivery models. Patient-led health technology approaches to lifestyle intervention may be instrumental to the future of health service design. Recruitment in this study will be limited to patients referred to dietetic services as part of their usual tertiary hospital outpatient care, and results may thus lack generalizability to other settings. The results will be broadly disseminated (locally, nationally and internationally) through established networks of the investigator team. There will be no restrictions placed on publication of any results. There is no intention for the use of professional writers. All authors will meet journal requirements for authorship.

## Appendix

Multimedia Appendix 1 Biochemical analytes.

Multimedia Appendix 2 Detailed description of telehealth exercise intervention.

## Abbreviations

5xSTS: repeated chair stand test
6MWT: 6-Minute Walk Test
AEP: accredited exercise physiologist
ANCOVA: analysis of covariance
APD: accredited practicing dietitian
DASH: dietary approaches to stop hypertension
FACIT: Functional Assessment of Chronic Illness Fatigue
HR: heart rate
HRR: heart rate reserve
IPAQ-SF: International Physical Activity Questionnaire Short Form
MET: metabolic equivalent
MetS: metabolic syndrome
MetSSS: Metabolic Syndrome Severity Score
MNMQ: Modified Nordic Musculoskeletal Questionnaire
PSQI: Pittsburgh Sleep Quality Index
RiR: repetitions in reserve
SAE: serious adverse event
SMART: specific, measurable, achievable, realistic, timely

## Data Availability

After completion and publication of the trial results, data requests can be submitted to the researchers at the Department of Nutrition and Dietetics at the Princess Alexandra Hospital, Brisbane, Australia.
