# Supplementary material for: Utilizing Technology for Diet and Exercise Change in Complex Chronic Conditions Across Diverse Environments (U-DECIDE): Protocol for a Randomized Controlled Trial
Source: JMIR Res Protoc. 2022 Jul 28;11(7):e37556. doi: 10.2196/37556 (PMC9377441; doi:10.2196/37556)
Supplement: Multimedia Appendix 1 [file resprot_v11i7e37556_app1.docx]

**Multimedia Appendix 2:**

**Blood biochemical analytes taken prior to baseline and end-of-program assessments**

| Blood Analytes |
| --- |
| Haemoglobin, Sodium, Potassium, Chloride, Bicarbonate, Glucose, Urea, Creatinine, Estimated Glomerular Filtration Rate, Albumin, Alkaline Phosphatase, Gamma-Glutamyl Transferase, Alanine Transaminase, Aspartate Transaminase, Calcium, Phosphate, Magnesium, Glycated Haemoglobin (HbA1c), Parathyroid Hormone, Vitamin D, C-reactive Protein, Total Cholesterol, Triglyceride, High-Density Lipoprotein Cholesterol, Low-Density Lipoprotein Cholesterol |
