# Supplementary material for: Utilizing Technology for Diet and Exercise Change in Complex Chronic Conditions Across Diverse Environments (U-DECIDE): Protocol for a Randomized Controlled Trial
Source: JMIR Res Protoc. 2022 Jul 28;11(7):e37556. doi: 10.2196/37556 (PMC9377441; doi:10.2196/37556)
Supplement: Multimedia Appendix 2 [file resprot_v11i7e37556_app2.docx]

**Multimedia Appendix 2:**

**EXERCISE INTERVENTION DETAILS**

**Exercise Monitoring**

Participants from both groups will be provided with a Fitbit Inspire HR (Fitbit, Inc., San Francisco, CA) and accompanying mobile app software during the baseline assessment. This will allow monitoring of physical activity, sedentary behaviour and exercise by the participants and the study investigators. Each participant will have a Fitbit account established with a study specific email address that will allow study staff access. This will permit appropriate real-time data extraction for the study. The AEP will assist the participant with initial account/device set-up and provide verbal and written instructions on how to use the device. The aerobic exercise prescription will require participants to exercise at a moderate-vigorous intensity (40-89% of Heart Rate Reserve (%HRR)) [1]. Age-Predicted Maximal Heart Rate (APMHR) will be calculated as 208 – 0.7*age [46]. APMHR for participants on beta-blockers will be calculated as 168 – 0.51*age [47]. The Fitbit allows for this range to be pre-set in a custom heart rate zone on the Fitbit mobile app. Participants can view their heart rate on the device screen during exercise. Participants can also see the amount of time spent per day in their custom heart rate zone, through the mobile app. As comfort of speech is an indicator of exercise intensity [2], the talk test will also be utilized to track exercise intensity. Participants will be asked to exercise between an intensity where they can talk but not sing (moderate intensity) and where they cannot say more than a few words without pausing (vigorous intensity) during each session. This will correlate to a rating of 3-7 (‘moderate’ to ‘severe’) on the CR10 Borg rating of perceived exertion scale (RPE) [3]. Both participant and observer RPE will be utilized to validate the custom heart rate zones to ensure both moderate and vigorous intensity physical activity are being met. New heart rate zones will be calibrated on an individual basis if intensity levels are not being subjectively met.

**Exercise Intervention**

Participants opting into either of the two exercise options will be asked to achieve a minimum of 150 minutes of moderate-vigorous intensity aerobic exercise, and 2 x 30 minute resistance exercise sessions per week, totalling a minimum of 210 minutes of structured exercise per week [4]. At least two days per week are to include resistance exercise. In the group based exercise option, the telehealth portion of the intervention will take place utilizing videoconferencing technology to deliver sessions to participants remotely. Sessions will be facilitated via the Queensland Health video portal in a group style format. Up to six participants will exercise with the AEP in each session. Prior to the commencement of the sessions, each participant will be provided with equipment (resistance bands with light and medium resistance grades) to facilitate remote aerobic and resistance exercise. The participants who opt into telehealth exercise sessions will be encouraged to attend one videoconference session per week for 26 weeks.

For both exercise options, prescription for the sessions will consist of 20 minutes of aerobic exercise and 30 minutes of resistance exercise with the provided equipment, whilst incorporating a five minute warmup/check-in and cooldown. The repetitions in reserve (RiR) method will be utilized for prescribing resistance exercise intensity. RiR refers to the estimated number of additional repetitions a participant believes they could have completed with the correct technique. An RiR=0 means that another repetition could not have been completed. Moderate intensity is between 1-4 RiR [50]. Resistance exercise will consist of 2-4 sets of a load between 1-4 RiR utilising the provided equipment. The AEP will make use of continual participant feedback to alter the resistance program as needed and take account of any adverse issues encountered during the intervention (e.g. musculoskeletal issues). Resistance exercise will be progressed over the course of the intervention. Varying the exercise prescription (e.g. increasing intensity and time) will be used to progressively overload the participants throughout the weeks. A minimum of 48 hours will be recommended between the resistance exercise sessions to promote adequate muscular recovery.

In both exercise options, the Physitrack app (Physitrack Ltd, London, UK) will be used to prescribe appropriate unsupervised home exercise for participants. Physitrack is a mobile app for exercise prescription and adherence monitoring, providing participants with video and text-based instructions for completing various forms of exercise whilst unsupervised. The app allows participants ask questions using the in-built ‘chat’ feature and allows the AEP to attach educational material to programs. The AEP will design home-based programs based on individual participant functional capacity, before prescribing them for unsupervised completion. To ensure safety, the AEP will not prescribe any exercise that has not been deemed appropriate to undertake unsupervised.

**Multimedia Appendix 1 references**

1. American College of Sports Medicine. ACSM's guidelines for exercise testing and prescription: Lippincott Williams & Wilkins; 2013
2. Reed JL, Pipe AL. The talk test: a useful tool for prescribing and monitoring exercise intensity. Curr Opin Cardiol. 2014;29(5):475-80. <https://doi.org/10.1097/HCO.0000000000000097>
3. Borg G. Borg's perceived exertion and pain scales: Human kinetics; 1998.
4. Hordern MD, Dunstan DW, Prins JB, Baker MK, Singh MA, Coombes JS. Exercise prescription for patients with type 2 diabetes and pre-diabetes: a position statement from Exercise and Sport Science Australia. J Sci Med Sport. 2012;15(1):25-31. <https://doi.org/10.1016/j.jsams.2011.04.005>
